# Shear Strength of Ultra-High-Performance Concrete (UHPC) Beams without Transverse Reinforcement: Prediction Models and Test Data

**DOI:** 10.3390/ma15144794

**Published:** 2022-07-08

**Authors:** Antony Kodsy, George Morcous

**Affiliations:** Durham School of Architectural Engineering and Construction, University of Nebraska-Lincoln, 1110 S. 67th Street, Omaha, NE 68182-0816, USA; gmorcous2@unl.edu

**Keywords:** UHPC, shear strength, prediction models, test data

## Abstract

The use of Ultra-High-Performance Concrete (UHPC) in beams has been growing rapidly in the past two decades due to its superior mechanical and durability properties compared to conventional concrete. One of the areas of interest to designers is the elimination of transverse reinforcement as it simplifies beam fabrication/construction and could result in smaller and lighter beams. UHPC has a relatively high post-cracking tensile strength due to the presence of steel fibers, which enhance its shear strength and eliminate the need for transverse reinforcement. In this paper, UHPC shear test data were collected from the literature to study the effect of the following parameters on the shear strength of UHPC beams without transverse reinforcement: compressive strength, tensile strength, level of prestressing, longitudinal reinforcement ratio, and fiber volume fraction. Statistical analysis of test data indicated that level of prestressing and tensile strength are the most significant parameters for prestressed UHPC beams, whereas longitudinal reinforcement ratio and tensile strength are the most significant parameters for non-prestressed UHPC beams. Additionally, shear strength of the tested UHPC beams was predicted using five models: RILEM TC 162-TDF, 2003, *fib* Model Code, 2010, French Standard NF P 18-710, 2016, PCI-UHPC Structures Design Guide, 2021, and Draft of AASHTO Guide Specification for Structural Design with UHPC, 2021. Comparing measured against predicted shear strength indicated that the French Standard model provides the closest prediction with the least scatter, where the average measured-to-predicted strength was 1.1 with a standard deviation of 0.38. The Draft of AASHTO provided the second closest prediction where the average measured-to-predicted strength was 1.3 with a standard deviation of 0.64. The other three models underestimated the shear strength.

## 1. Introduction

Ultra-high-performance concrete (UHPC) is a relatively new class of concrete that has superior mechanical and durability properties compared to conventional concrete. This is primarily due to its very dense matrix of particles and presence of high strength steel fibers. The stress-strain behavior of UHPC in tension and compression is significantly different than that of conventional concrete [1,2]. Limited experimental investigations were conducted to evaluate the shear capacity of UHPC beams when transverse reinforcement is removed. The elimination of transverse reinforcement greatly simplifies beam fabrication and construction and results in thinner webs and lighter sections. The addition of steel fibers in UHPC beams significantly increases the post-cracking tensile strength that typically controls the shear strength in beams (i.e., diagonal tension). Shear strength of UHPC is influenced by several parameters, such as fiber content, concrete compressive and tensile strengths, longitudinal reinforcement ratio, and level of prestressing. Currently, there are limited prediction models that estimate the shear strength of UHPC beams; moreover, there are relatively high margins of safety used in these models due to the limited amount of shear test data [3,4].

This paper presents a database of UHPC beam shear tests available in the literature to study the effect of various parameters on the shear strength when transverse reinforcement is eliminated. Then, five of the popular prediction models, namely RILEM TC 162-TDF, 2003 [5], *fib* Model Code, 2010 [6], French Standard NF P 18-710, 2016 [7], PCI-UHPC Structures Design Guide, 2021 [8], and Draft of AASHTO Guide Specification for Structural Design with UHPC, 2021 [9] are evaluated by comparing measured against predicted shear strength to determine their accuracy and consistency. The work done in this study is considered the first attempt to collect test data of UHPC beams without shear reinforcement failing in diagonal tension mode.

## 2. Materials and Methods

This section presents an overview on the UHPC beam shear prediction models and the experiments done on prestressed and non-prestressed UHPC beams.

### 2.1. Prediction Models

Five prediction models are presented in this section: RILEM TC 162-TDF, 2003 [5], *fib* Model Code, 2010 [6], French standard, NF P 18-710, 2016 [7], PCI-UHPC Structures Design Guide, 2021 [8], and Draft of AASHTO Guide Specification for Structural Design with UHPC, 2021 [9]. The first three models represent the historical evolution of shear strength prediction approaches from conventional concrete to fiber-reinforced concrete to UHPC internationally, whereas the last two models are recently published in the United States to promote the implementation of UHPC in structural applications. The main differences among these models are the terms that account for the post-cracking tensile strength of UHPC and safety factors.

#### 2.1.1. RILEM TC 162-TDF, 2003

The prediction model of shear strength of conventional concrete beams given in the Eurocode 2 part 1, 1991 [10] was used as the main framework in developing this prediction model [11]. Eurocode 2 only considers the pre-peak behavior of conventional concrete in tension, whereas the effect of steel fibers on the post-peak behavior of fiber-reinforced concrete is considered in this model. This leads to the consideration of the tensile stress-strain behavior of fiber-reinforced concrete in this model. The ultimate shear resistance of a section is estimated as the algebraic sum of contributions of concrete ($V_{cd}$), stirrups and/or inclined bars, and steel fibers ($V_{fd}$). Fiber contribution is estimated according to the following equation:

(1) $$V_{fd}=0.7\;k_{f}k\;b_{w}\;d\;{Ʈ}_{fd}\;\left[N\right]$$

where $k_{f}$ is a factor that accounts for the flanges contribution in T-shaped sections and recommended to be taken as 1.0 for other shapes. Flange contribution is typically neglected in most shear strength prediction models for simplicity and due to the fact that diagonal tension failure starts at the web of flanged sections. $k_{f}$ is calculated as follows:

(2) $$k_{f}=1+n\;\left(\frac{h_{f}}{b_{w}}\right)\left(\frac{h_{f}}{d}\right)$$

(3) $$n=\frac{b_{f}-b_{w}}{h_{f}}\;\leq 3\;\mathrm{and}\;n\;\leq \;\frac{3\;b_{w}}{h_{f}}$$

where $b_{f}$ is the flanges width [mm]; $b_{w}$ is the minimum cross-section web width [mm] over d [mm]; d is the effective depth of the section [mm]; $h_{f}$ is the flanges height [mm]; k is a factor to account for the size of the section taken as 1 + $\sqrt{\frac{200}{d}}$ ≤ 2; ${Ʈ}_{fd}$ is the design value of increase in shear capacity due to steel fibers taken as ($0.12\;f_{R,4}$) [MPa]; $f_{R,4}$ is the residual flexural tensile strength of the prism corresponding to the crack mouth opening displacement (CMOD) of 3.5 mm [MPa]. Residual flexural tensile strengths ($f_{R,i}$) are estimated by a three-point bending test on a 150 × 150 × 550 mm notched prism. The stress versus strain behavior is obtained from the load-deflection or load-CMOD relation of the prism. The load-CMOD curve is defined using four points (i = 1 through 4) that corresponds to the CMOD of 0.5, 1.5, 2.5, and 3.5 mm, respectively. $f_{R,i}$ is calculated as follows:

(4) $$f_{R,i}=\frac{3\;F_{R,i}\;x\;L}{2b\;x\;h_{sp}{}^{2}}\;\left[\mathrm{MPa}\right]$$

where $F_{R,i}$ is the measured load at (CMODi) [N]; L is the prism span [mm]; b is the prism width [mm]; $h_{sp}$ is the depth between the notch tip to the extreme compression fibers of the prism cross-section [mm].

The concrete contribution is calculated according to Equation (5).

(5) $$V_{cd}=\left[0.12\;k\;{\left(100\;\rho_{1}\;f_{ck}\right)}^{\frac{1}{3}}+0.15\;{Ϭ}_{cp}\right]\;b_{w}\;d\;\left[N\right]$$

(6) $$\rho_{1}=\frac{A_{l}}{b_{w}\;d}$$

(7) $${Ϭ}_{cp}=\frac{N_{sd}}{A_{c}}\;\left[\mathrm{MPa}\right]$$

where $\rho_{1}$ is the ratio of longitudinal reinforcement (recommended not to be taken greater than 2%); $A_{l}$ is the area of tension reinforcement extending at least (d + anchorage length) beyond the considered section [mm^2^]; $f_{ck}$ is the cylinder characteristic compressive strength [MPa]; ${Ϭ}_{cp}$ is the factor to account for the level of axial loading or prestressing in the beam [MPa]; $N_{Sd}$ is the axial force in the section due to loading or prestressing (positive for compression) [N]; $A_{C}$ is the area of the beam cross-section [mm^2^].

#### 2.1.2. *Fib* Model Code, 2010

This prediction model is developed for steel fiber-reinforced conventional concrete and is not validated yet for UHPC. The approach bears some similarities to the RILEM TC 162-TDF, 2003 [5] prediction model with the exception of having the steel fibers contribution coupled with the concrete contribution in one term ($V_{Rd,F})$ as follows:

(8) VRd,F=0.18γc· k ·100·ρ11+7.5fFtukfctk·fck13+0.15 ·Ϭcp·bw·d [N]

where terms such as k, $\rho_{1}$, ${Ϭ}_{cp}$, $b_{w}$, and d are defined similar to RILEM TC 162-TDF, 2003 [5]; $\gamma_{c}$ is a partial safety factor for concrete with no fibers (recommended to be taken as 1.5); $f_{Ftuk}$ is the characteristic value of ultimate residual tensile strength calculated as follows:

(9) $$f_{Ftuk}=\frac{f_{R,3}}{3}\;\left[\mathrm{MPa}\right]$$

(10) $$f_{Ftuk}=f_{Fts}-\frac{w_{u}}{CMOD_{3}}\;\left(f_{Fts}-0.5\;f_{R,3}+0.2\;f_{R,1}\right)\geq 0\;\left[\mathrm{MPa}\right]$$

(11) $$f_{Fts}=0.45\;f_{R,1}\left[\mathrm{MPa}\right]$$

Similar to the RILEM TC 162-TDF, 2003 [5] model, $f_{R,1}$ and $f_{R,3}$ are the residual flexural tensile strengths corresponding to CMOD of 0.5 mm and 3.5 mm, respectively [MPa]; $f_{Fts}$ is the characteristic residual tensile strength (post-cracking strength at serviceability crack opening) [MPa]; $w_{u}$ is the maximum crack opening that is acceptable in structural design (recommended to be taken as 1.5 mm); $f_{Ftuk}$ is determined according to the rigid-plastic model Equation (9), or the linear model based on Equations (10) and (11); $f_{ctk}$ is the characteristic tensile strength of concrete containing no fibers [MPa] and can be determined as follows:

(12) $$f_{ctk}=2.12\ln\left(1+0.1\left(f_{ck}+8\;\mathrm{MPa}\right)\right)\;\left[\mathrm{MPa}\right]\;(for\;f_{ck}>50\;\mathrm{MPa})$$

where $f_{ck}$ is the cylinder characteristic compressive strength [MPa]. The residual flexural tensile strengths are determined experimentally according to the EN 14,651 [12], where a three-point bending test is performed on a 150 × 150 × 550 mm notched prism.

#### 2.1.3. French Standard, NF P 18-710, 2016

Based on the AFGC, 2013 [13], this model was developed and calibrated for UHPC. The basis of this model is similar to the AASHTO LRFD [14] simplified version of the Modified Compression Field Theory (MCFT) procedure, where the shear strength of a beam consists of a concrete contribution term ($V_{Rd,c}$), and a shear reinforcement contribution term ($V_{Rd,s}$). Then, the tensile strength of UHPC is represented by the addition of a fiber contribution term ($V_{Rd,f}$) [15]. The concrete contribution is calculated according to Equations (13) and (14) for prestressed and non-prestressed beam sections, respectively.

(13) $$V_{Rd,c}=\frac{0.24}{\gamma_{cf}\gamma_{E}}\;k\;f_{ck}{}^{1/2}\;b_{w}\;z\;\left[N\right]$$

(14) $$V_{Rd,c}=\frac{0.18}{\gamma_{cf}\gamma_{E}}\;k\;f_{ck}{}^{1/2}\;b_{w}\;h\;\left[N\right]$$

(15) $$k=1+\frac{3\;{Ϭ}_{cp}}{f_{ck}}\;,\;\;\mathrm{for}\;{Ϭ}_{cp}\;\geq \;0$$

**Figure 1:**
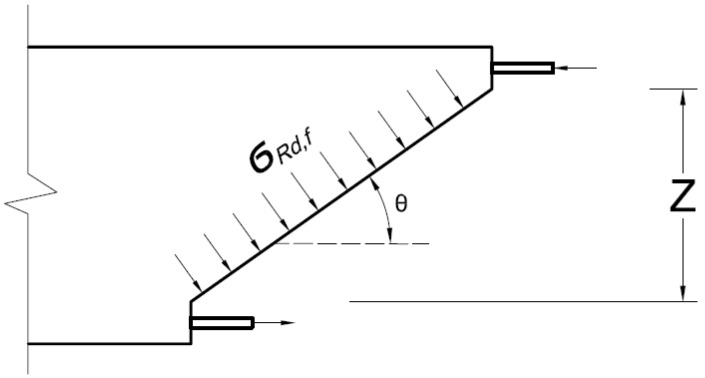
Tensile stresses carried by steel fibers in a beam cross-section according to the Modified Compression Field Theory [16].

The terms k, $f_{ck}$, $b_{w}$, and ${Ϭ}_{cp}$ are defined similarly to the terms in the previous models; The partial safety factors $\gamma_{cf}$ and $\gamma_{E}$ are recommended to be taken as 1.5 after being multiplied; $\gamma_{cf}$ is a factor for UHPC in tension recommended to be taken as 1.3, and $\gamma_{E}$ is a factor to account for the uncertainty in extrapolating the model developed for high performance concrete to UHPC; (z) is the lever arm of the internal moment in the cross-section (typically taken as 90% of the section depth). A larger number of test data is required to lower the safety factors used in the calculation of concrete contribution. The contribution term of fibers is calculated by quantifying the post-crack residual tensile strength resisting the main crack across the angle θ across z as shown in Figure 1 [16].

Fiber contribution term is calculated as follows:

(16) $$V_{Rd,f}=\frac{A_{fv}\;{Ϭ}_{Rd,f}}{\tan\left(\theta \right)}\;\left[N\right]$$

(17) $${Ϭ}_{Rd,f}=\frac{1}{K\;\gamma_{cf}}\;\frac{1}{w_{lim}}\;\int_{0}^{w_{lim}}{Ϭ}_{f}\left(w\right)\cdot dw\;\left[\mathrm{MPa}\right]$$

where $A_{fv}$ is the area where the fibers are effective ($A_{fv}=b_{w}\;z$) [mm^2^]; ${Ϭ}_{Rd,f}$ is the residual tensile strength of the fiber-reinforced member [MPa]; θ is the angle between the principal compression stress and beam axis in degrees, and is recommended not to be taken less than 30 degrees; ${Ϭ}_{Rd,f}$ is estimated as the area under the stress-crack width curve of a 3-point bending test; K is a reduction factor to account for the difference between fibers orientation of the prism and that of the structure; the K factor is recommended to be taken between 1.0 to 1.4; $w_{lim}$ is the maximum crack width reached at the ultimate bending moment, or the admissible crack width (recommended as 0.3 mm). The dimensions of the flexure test prism depend on the length of fibers (l_f_). For (l_f_ ≤ 15 mm), 70 × 70 × 280 mm prisms are recommended, and for (15 mm < l_f_ ≤ 20 mm), 100 × 100 × 380 mm prisms are recommended to be used. The depth of the notch is equal to 10% of the prism height to allow for an efficient localization of the crack, and to minimize the risk of cracking outside the notch location. The distance between the two bearing points must be three times the depth of the prism. The residual tensile strength can be also calculated from direct tension tests on un-notched prisms.

#### 2.1.4. PCI-UHPC Structures Design Guide, 2021

The Precast/Prestressed Concrete Institute (PCI) recently published Phase II report for the implementation of using UHPC in precast bridges and buildings. The report provides guidelines for the design of UHPC members, as well as acceptance criteria for PCI-UHPC material mechanical properties and production. The model presented in the report is based on the MCFT and A AASHTO LRFD [14]. The ultimate shear load carrying capacity ($V_{n}$) is taken as the sum of the contributions of UHPC tensile strength ($V_{cf}$), shear reinforcement ($V_{s}$), and the component of prestressing force resisting vertical shear ($V_{p}$). $V_{cf}$ is calculated according to the following equation:

(18) $$V_{cf}=1.33\;f_{rr}\;b_{w}\;d\;\cot\left(\theta \right)\;\left[N\right]$$

where $f_{rr}$ is the residual tensile strength of UHPC and recommended to be taken as 5.2 MPa for UHPC meeting minimum PCI-UHPC tensile properties requirements; this value was calculated based on the minimum required peak flexural strength according to ASTM C1609 [17] of 13.8 MPa multiplied by a 0.375 conversion factor according to [18,19]. Other parameters are similar to what was described in the previous models. The crack angle is estimated according to the following equations:

(19) $$\theta =29+3500\;{Ꜫ}_{s}$$

where ${Ꜫ}_{s}$ is the strain at the level of tension reinforcement and calculated as follows for positive values of ${Ꜫ}_{s}$:

(20) $${Ꜫ}_{s}=\frac{\left(\frac{M_{u}}{d}\right)+\left(V_{u}-V_{p}\right)-P_{e}}{\left(E_{s}A_{s}+E_{p}A_{ps}\right)}\leq 0.006$$

where $M_{u}$ and $V_{u}$ are the applied factored moment and vertical shear at the critical section under consideration; $P_{e}$ is the effective axial prestressing force acting on the section; $E_{s}$ and $E_{p}$ are the moduli of elasticity of reinforcing and prestressing steel, respectively; $A_{s}$ and $A_{ps}$ are the area of reinforcing and prestressing steel, respectively. For negative values of ${Ꜫ}_{s}$ the equation becomes as follows:

(21) $${Ꜫ}_{s}=\frac{\left(\frac{M_{u}}{d}\right)+\left(V_{u}-V_{p}\right)-P_{e}}{\left(E_{s}A_{s}+E_{p}A_{ps}+E_{c}A_{ct}\right)}\geq -0.0004$$

where $E_{c}$ is the UHPC modulus of elasticity, and $A_{ct}$ is the area of UHPC on the flexural tension side of the member measured from the mid-height of the section. The upper and lower bounds of ${Ꜫ}_{s}$ are selected to provide an angle between 27.6 and 50.0 degrees. It should be noted that this model is based on the load and resistance factor design which utilizes a strength reduction factor and a load magnification factor. Strength reduction factors were not considered when the shear strength was predicted using the model in that paper. Furthermore, the model assumes proper reinforcement is provided and developed in the flexural tension side of the critical section to achieve the full diagonal tension strength.

#### 2.1.5. Draft of AASHTO Guide Specification for Structural Design with UHPC, 2021

A draft guide specification for the design of concrete elements fabricated with UHPC is currently being considered by the AASHTO CBS T-10 committee to be included in the next revision of the AASHTO LRFD [14]. The document was developed by the Federal Highway Administration (FHWA) Turner-Fairbank Highway Research Center. Similar to the French standard [7] and AASHTO LRFD [14], the model is based on the MCFT with the analysis of the principal strains at critical sections. The nominal shear resistance of a member ($V_{n}$) is taken as the sum of the contributions of UHPC tensile strength ($V_{UHPC}$), shear reinforcement ($V_{s}$), and component of prestressing force resisting vertical shear ($V_{p}$). $V_{UHPC}$ is calculated according to the following equation:

(22) $$V_{UHPC}=\gamma \;f_{t,loc}\;b_{w}\;d\;\cot\left(\theta \right)\;\left[N\right]$$

where γ is a reduction factor to account for the variability of tensile stresses carried by UHPC (recommended not to exceed 0.85), $f_{t,loc}$ is the localization tensile strength of UHPC estimated by means of direct tension testing on 50 × 50 mm^2^ prisms, and other parameters are similar to what was described before. The crack angle $\left(\theta \right)$ in this model is limited to a range from 25 to 45 degrees, and is estimated according to the following equations:

(23) $${Ꜫ}_{t,loc}=\frac{{Ꜫ}_{s}}{2}\left(1+\cot^{2}\theta \right)+\frac{2f_{t,loc}}{E_{c}}\cot^{4}\theta +\frac{2\rho_{v}f_{v}}{E_{c}}\sin\alpha {\;\cot}^{2}\theta \;[1+\cot^{2}\theta +\cot\alpha (\tan\;\theta +\cot\theta )]$$

(24) $${Ꜫ}_{2}=-\frac{2f_{t,loc}}{E_{c}}{\;\cot}^{2}\theta -\frac{2\rho_{v}f_{v}}{E_{c}}\sin\alpha \;[1+\cot^{2}\theta +\cot\alpha (\tan\;\theta +\cot\theta )]$$

(25) $${Ꜫ}_{v}={Ꜫ}_{t,loc}-0.5\;{Ꜫ}_{s}+{Ꜫ}_{2}$$

(26) $$f_{v}=E_{s}\;{Ꜫ}_{v}\;\leq \;f_{y}$$

where ${Ꜫ}_{t,loc}$ is the localization strain obtained by the direct tension testing when the tensile stresses carried by the UHPC prism start to decrease consistently, and recommended to be taken between 0.004 to 0.010 [20,21,22]; ${Ꜫ}_{s}$ is longitudinal strain at the level of reinforcement calculated as follows:

(27) $${Ꜫ}_{s}=\frac{\frac{\left|M_{u}\right|}{d_{v}}+0.5\;N_{u}+\left|V_{u}-V_{P}\right|-A_{ps}\;f_{po}-\gamma \;f_{t,loc}\;A_{ct}}{E_{s}\;A_{s}+E_{p}\;A_{ps}}$$

where $\left|M_{u}\right|$ is the absolute value of the factored moment at the design section, not to be taken as less than $\left|V_{u}-V_{P}\right|d_{v}$; $N_{u}$ is the factored axial force at the design section, taken as positive if tension and negative if compression; $V_{u}\;$ is the factored shear force at the design section; $A_{ps}$ and $A_{s}$ are the area of prestressing steel and non-prestressed steel, respectively, in the flexural tension side of the member; $f_{po}$ is the parameter taken as modulus of elasticity of prestressing steel multiplied by the locked-in difference in strain between the prestressing steel and surrounding UHPC and could be taken as 70% of the ultimate tensile strength of the strands for appropriate levels of prestressing; $A_{ct}$ is the area of UHPC in the flexural tension side of the member; $E_{s}$ and $E_{p}$ are the moduli of elasticity of non-prestressed and prestressing steel respectively; $\rho_{v}$ is the transverse reinforcement ratio calculated as the area of transverse reinforcement divided by bar spacing and web width; $f_{v}$ is the stress in transverse reinforcement; ${Ꜫ}_{2}$ is the diagonal compressive strain in the section; and ${Ꜫ}_{v}$ is the vertical strain in transverse reinforcement at the design section. Similar to the PCI-UHPC model, this model is based on the load and resistance factor design and the model also assumes proper reinforcement is provided and developed in the flexural tension side of the critical section.

### 2.2. Shear Experiments

**Figure 2:**
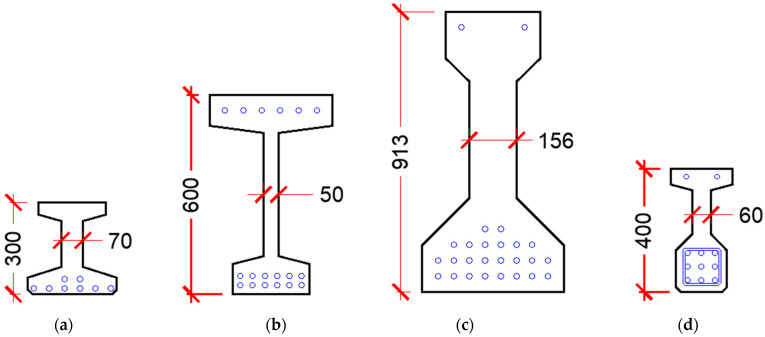

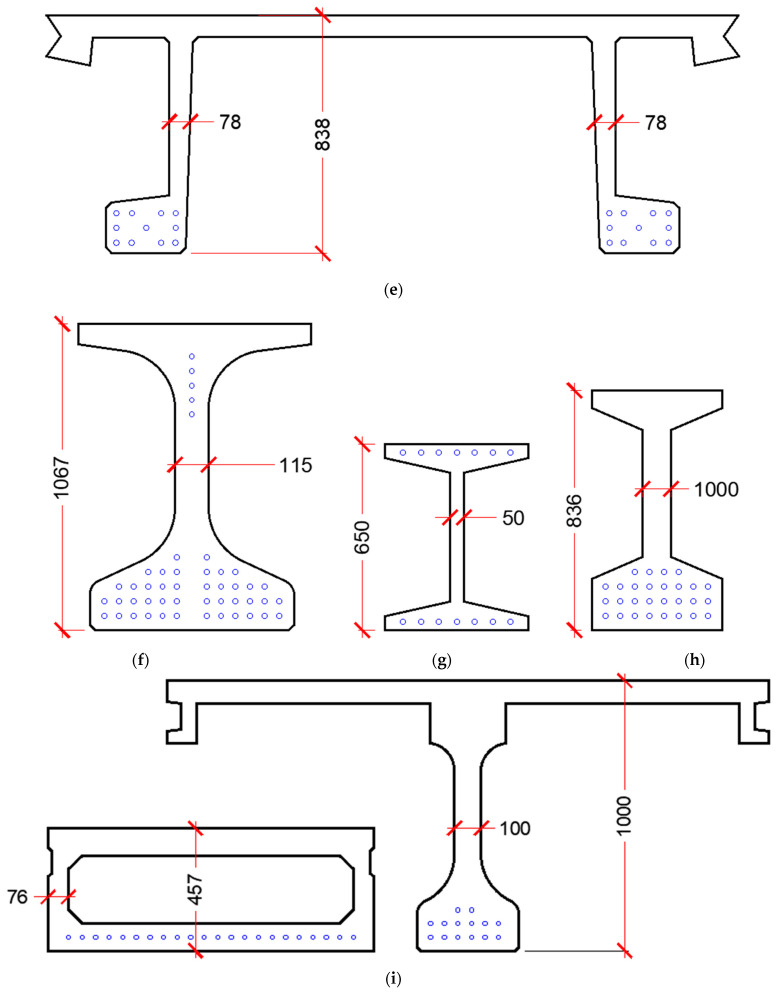

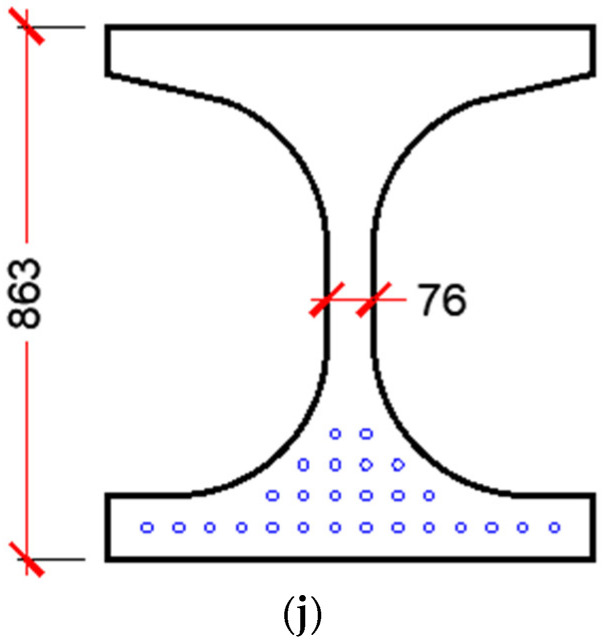
Girder cross-sections of the shear test data of prestressed beams (units in millimeter). (**a**) Hegger et al., 2004 [24]. (**b**) Voo et al., 2006 [23]. (**c**) Graybeal, 2006 [27]. (**d**) Hegger and Bertram, 2008 [25]. (**e**) Graybeal, 2009 [28]. (**f**) Wipf et al., 2009 [29]. (**g**) Voo et al., 2010 [30]. (**h**) Crane, 2010 [15]. (**i**) Tadros et al., 2021 [8]. (**j**) Tadros et al., 2021 [8].

**Figure 3:**
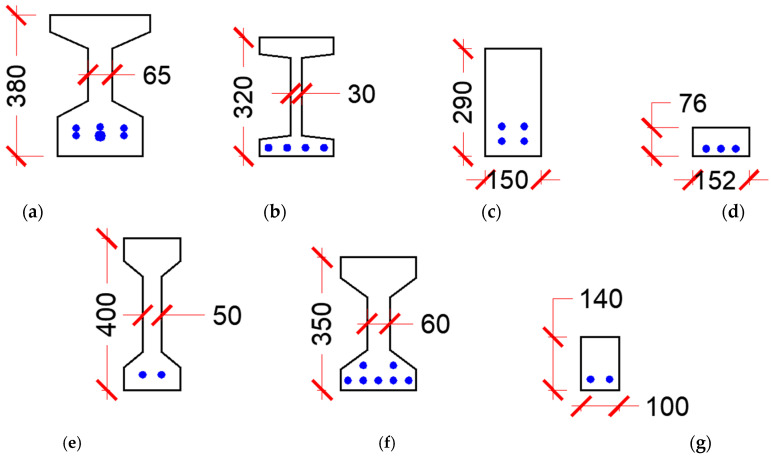
Girder cross-sections of the shear test data of non-prestressed beams (units in millimeter). (**a**) Baby et al., 2010 [31,32]. (**b**) Fehling et al., 2012 [33]. (**c**) Lim et al., 2016 [26]. (**d**) Pourbaba et al., 2018 [34,35]. (**e**) Pansuk et al., 2017 [36]. (**f**) Meszoly et al., 2018 [37]. (**g**) Ridha et al., 2018 [38].

Table A1 and Table A2 present a summary of the shear experiments conducted on prestressed and non-prestressed UHPC beams, respectively. All test data were conducted on beams without transverse reinforcement. Figure 2 shows a schematic of the cross-sections of the prestressed beams, whereas Figure 3 shows that of non-prestressed beams. This data was collected from 16 research programs conducted on UHPC beams reinforced longitudinally with mild reinforcement (yield strength ranging from 400 to 600 MPa or prestressing strands (tensile strength ranging from 1700 to 1860 MPa). All UHPC mixes in these experiments had straight fibers that have a tensile strength ranging from 1800 to 2600 MPa, except for Voo et al., 2006 [23] who used a mix of straight- and end-hooked fibers in some tests. 

Hegger et al., 2004 [24], performed one test on an I-shaped beam to investigate the bond anchorage behavior and shear strength of the section. Some slippage occurred at the bottom strands and the utilization of the prestressing force was nearly 80% at failure. Voo et al., 2006 [23] conducted seven tests where the combined fiber volume fraction was 2.5% for all beams except SB4 which had 1.25%. The used fibers were a combination of straight- (type I) and end-hooked (type II) types. Specimens SB1, SB2, and SB3 only contained type I fibers, whereas specimens SB4, SB5, and SB7 contained 1.25%, 1%, 0.62% type II fibers, respectively, and specimen SB6 contained only type II fibers. Hegger and Bertram, 2008 [25], conducted shear tests on prestressed I-shaped beams to estimate the bond anchorage of strands and the shear capacity of the section with and without openings in the web. Five beams without web openings were only considered in this study.

Crane, 2010 [15], conducted six tests on bulb-tee girders having a depth of 835 mm, and a 200 mm cast in place of a high-performance concrete deck having a compressive strength of 84 MPa was placed on the beams. Because of the significant differences in concrete properties between the deck and UHPC girder, the effective shear depth was based only on the girder and not the composite section. Lim et al., 2016 [26], conducted shear tests on rectangular beams and the primary test variable was the shear reinforcement ratio. Only one specimen (out of four) that did not contain any shear reinforcement and failed in diagonal tension was included in this study.

All the specimens had a shear span to depth ratio of at least 2.3 and experienced a diagonal tension failure. Diagonal tension failure in UHPC beams typically starts with several narrow and closely spaced cracks parallel to the failure crack angle. One of these cracks starts to grow wider and longer with the increase of load until failure [27]. The unreported crack angles (θ) were reasonably estimated based on failure photos, whereas post-cracking residual tensile strengths (${Ϭ}_{Rd,f}$) were reasonably estimated based on flexural or direct tension test results.

## 3. Results

This section presents the parametric study done on the shear experiments discussed earlier. Then comparison against prediction models is presented for the five models.

### 3.1. Shear Strength Parameters

**Figure 4:**
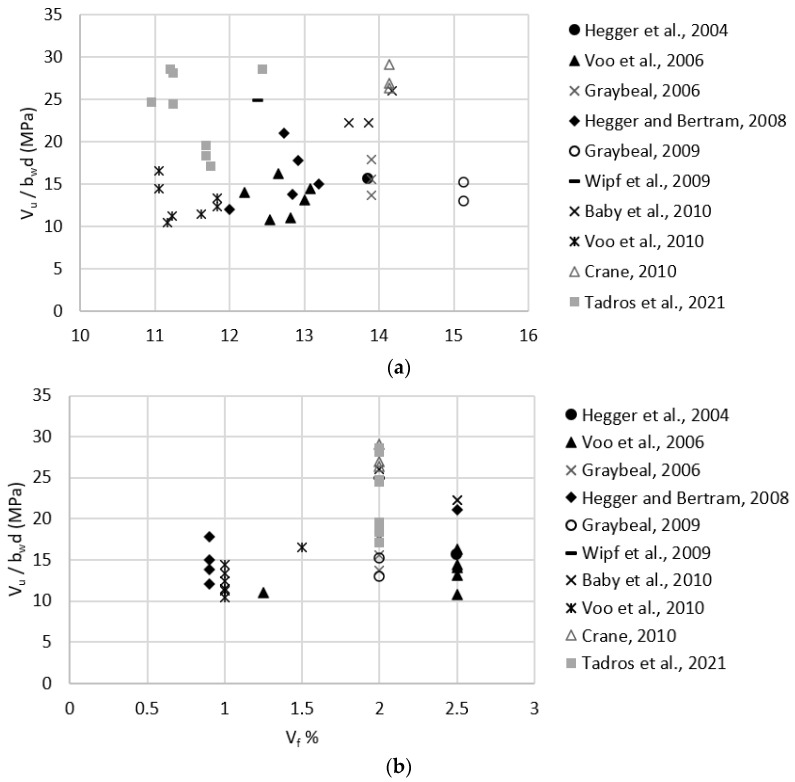

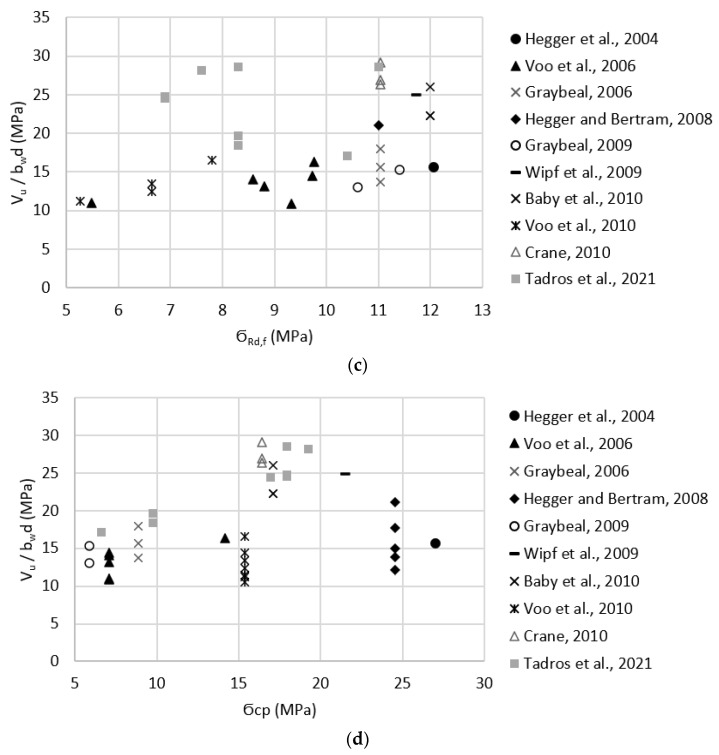
Effect of key parameters on shear strength of prestressed UHPC beams. (**a**) effect of the square root of UHPC compressive strength (${\left[{f_{c}}^{\prime }\right]}^{0.5}$). (**b**) effect of fiber volume fraction ($V_{f}$ ). (**c**) effect of UHPC post-cracking tensile strength (${Ϭ}_{Rd,f}$ ). (**d**) effect of level of prestressing (${Ϭ}_{cp}$ ).

Test data listed in Table A1 were used to evaluate the effect of key parameters, such as compressive strength, fiber content, tensile strength, and level of prestressing, on the shear strength of prestressed UHPC beams. Figure 4a–d plots ${\left[{f_{c}}^{\prime }\right]}^{0.5}$, $V_{f}$, ${Ϭ}_{Rd,f}$, and ${Ϭ}_{cp}$, respectively, versus measured shear strength. These plots indicate there is no strong correlation between any of these parameters and the shear strength of prestressed UHPC beams. Correlation coefficients were calculated using the Pearson correlation test and were found to be 0.05, 0.36, 0.44, and 0.33 for ${\left[{f_{c}}^{\prime }\right]}^{0.5}$, $V_{f}$, ${Ϭ}_{Rd,f}$, and ${Ϭ}_{cp}$, respectively. These coefficients indicate that there is only a moderate correlation between ${Ϭ}_{Rd,f}$ and the shear strength, which is in agreement with the prediction models presented earlier in Section 2. In addition, to test for the statistical significance of these key parameters on the shear strength of prestressed beams, a multiple regression analysis was performed for shear strength as the dependent variable and ${\left[{f_{c}}^{\prime }\right]}^{0.5}$, $V_{f}$, ${Ϭ}_{Rd,f}$, and ${Ϭ}_{cp}$ as the independent variables using 5% significance level. Only ${Ϭ}_{Rd,f}$, and ${Ϭ}_{cp}$ were found to have significant effects as their *p*-values were 0.019, and 0.001, respectively, whereas ${\left[{f_{c}}^{\prime }\right]}^{0.5}$ and $V_{f}$ had *p*-values of 0.43, and 0.69, respectively, which indicate that their effects are statistically insignificant.

**Figure 5:**
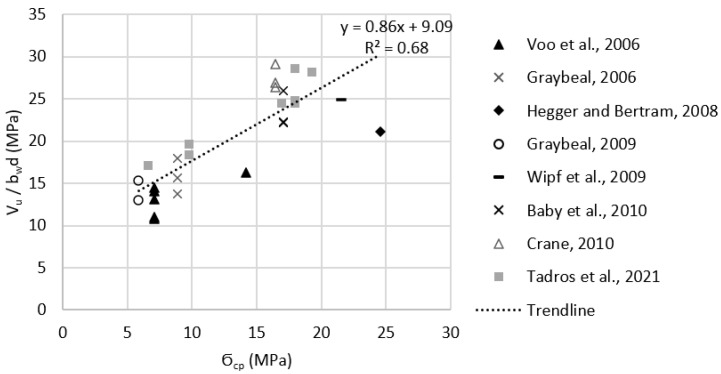
Effect of the level of prestressing (${Ϭ}_{cp}$) on the shear strength for prestressed beam with $V_{f}$ ≥ 2.0%.

In order to have a more homogenous group of test data, specimens with a fiber volume fraction less than 2% ($V_{f}$ < 2.0%) were omitted since the commonly used $V_{f}$ in UHPC mixtures is at least 2% [39]. In addition, the Hegger et al., 2004, specimen was omitted as the prestressing force was not fully utilized due to strand slippage [24]. The remaining specimens yielded a strong correlation between level of prestressing (${Ϭ}_{cp})$ and the shear strength (*V_u_/b_w_d*) as shown in Figure 5. Multiple regression analysis was done on that group and resulted in ${Ϭ}_{cp}$ and $V_{f}$ being statistically significant as their *p*-values were 1 × 10^−7^, and 6 × 10^−5^, respectively, whereas ${\left[{f_{c}}^{\prime }\right]}^{0.5}$ and ${Ϭ}_{Rd,f}$ had *p*-values of 0.96, and 0.86, respectively. The resulting relation between the shear strength and $V_{f}$ was very weak which is in agreement with the Pearson correlation test results of the multiple regression analysis. The standard error of the model containing all data points was 3.56, whereas the standard error value for the ($V_{f}$ > 2.0%) group was 2.90. This indicates that the ($V_{f}$ > 2.0%) regression model provides higher accuracy than model containing all data points.

**Figure 6:**
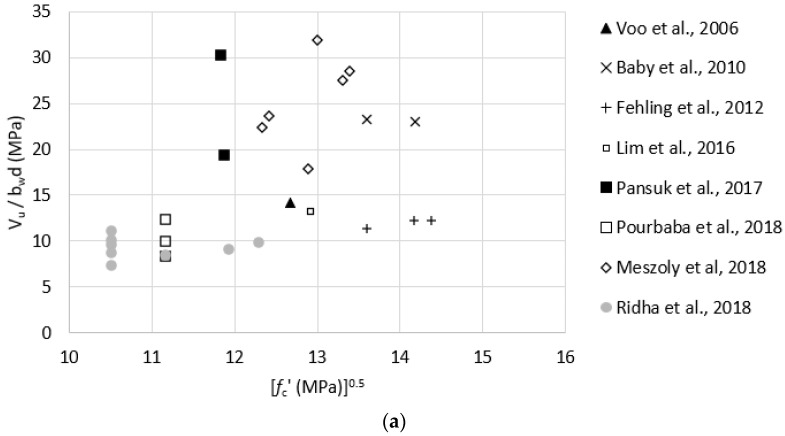

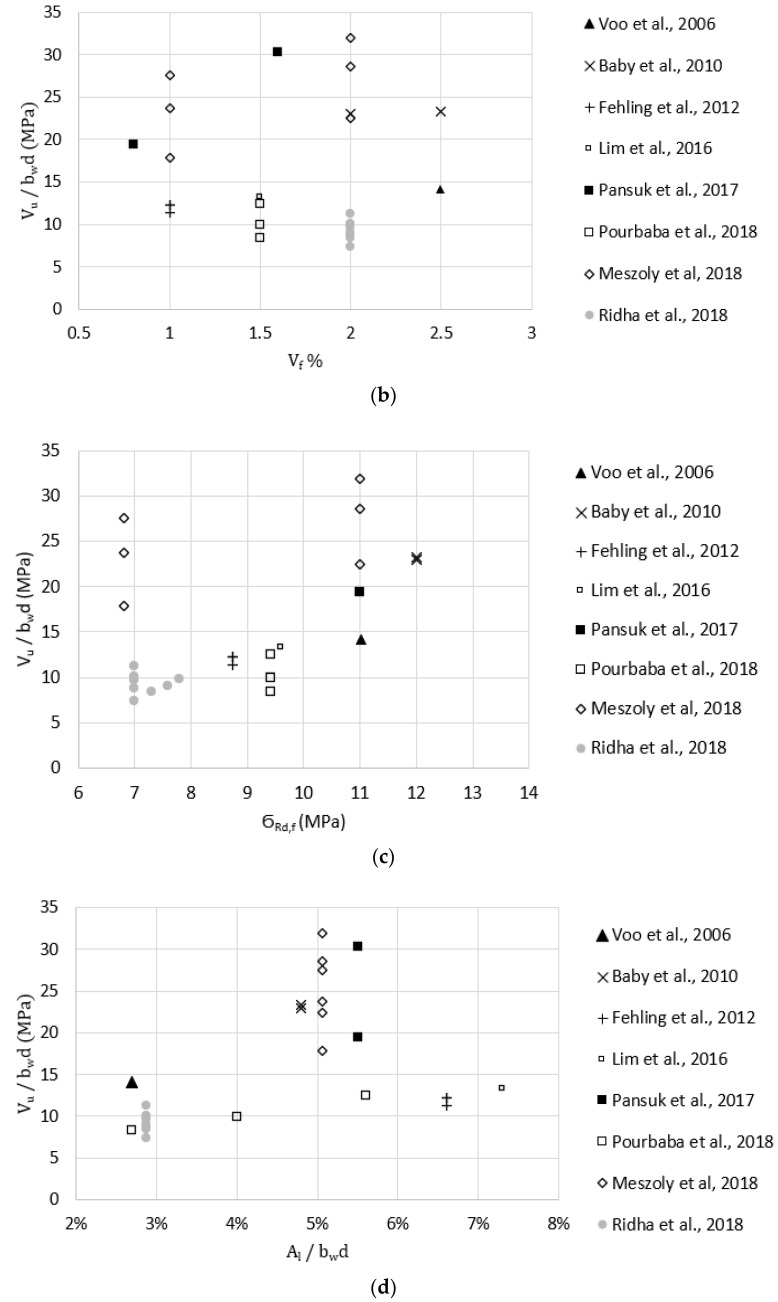
Effect of key parameters on shear strength of non-prestressed UHPC beams. (**a**) effect of the square root of UHPC compressive strength (${\left[{f_{c}}^{\prime }\right]}^{0.5}$). (**b**) effect of fiber volume fraction ($V_{f}$ ). (**c**) effect of UHPC post-cracking tensile strength (${Ϭ}_{Rd,f}$ ). (**d**) effect of longitudinal reinforcement ratio ($A_{l}/b_{w}d$).

Test data listed in Table A2 were used to evaluate the effect of key parameters, such as compressive strength, fiber content, tensile strength, and reinforcement ratio, on the shear strength of non-prestressed UHPC beams. Figure 6a–d plots ${\left[{f_{c}}^{\prime }\right]}^{0.5}$, $V_{f}$, ${Ϭ}_{Rd,f}$, and $A_{l}/b_{w}d$, respectively, versus measured shear strength. These plots indicate no strong correlation between any of these parameters and the shear strength of non-prestressed UHPC beams. Correlation coefficients were calculated using the Pearson correlation test and were found to be 0.49, −0.05, 0.60, and 0.43 for ${\left[{f_{c}}^{\prime }\right]}^{0.5}$, $V_{f}$, ${Ϭ}_{Rd,f}$, and $A_{l}/b_{w}d$, respectively. These coefficients indicate that there is only a moderate correlation between ${Ϭ}_{Rd,f}$ and the shear strength, which is in agreement with the prediction models presented earlier in Section 2. In addition, to test for the statistical significance of these key parameters on the shear strength of non-prestressed beams, a multiple regression analysis was performed for shear strength as the dependent variable and ${\left[{f_{c}}^{\prime }\right]}^{0.5}$, $V_{f}$, ${Ϭ}_{Rd,f}$, and $A_{l}/b_{w}d$ as the independent variables using 5% significance level. Only ${Ϭ}_{Rd,f}$ was found to have a significant effect, where the *p*-value was 0.024, whereas ${\left[{f_{c}}^{\prime }\right]}^{0.5}$, $V_{f}$, and $A_{l}/b_{w}d$ had *p*-values of 0.22, 0.92, and 0.90, which indicate that their effects are statistically insignificant.

**Figure 7:**
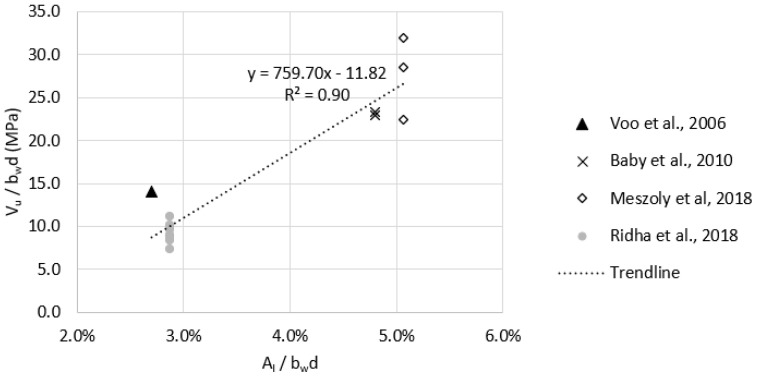
Effect of the longitudinal reinforcement ratio ($A_{l}/b_{w}d$) on the shear strength of non−prestressed UHPC beams with $V_{f}$ ≥ 2.0%.

In order to have a more homogenous group of test data, specimens with a fiber volume fraction of less than 2% ($V_{f}$ < 2.0%) were omitted since the commonly used $V_{f}$ in UHPC mixtures is at least 2% [39]. The remaining specimens yielded a strong correlation between reinforcement ration ($A_{l}/b_{w}d$) and the shear strength as shown in Figure 7. Multiple regression analysis was done on that group and resulted in $A_{l}/b_{w}d$ being statistically significant as the *p*-value was 0.024, whereas ${\left[{f_{c}}^{\prime }\right]}^{0.5}$, $V_{f}$, and ${Ϭ}_{Rd,f}$ had *p*-values of 0.82, 0.89, and 0.49, respectively, which is in agreement with the Pearson correlation test results. The standard error of the model containing all data points was 6.27, whereas the standard error value for the ($V_{f}$ > 2.0%) group was 2.80. This indicates that the ($V_{f}$ > 2.0%) regression model provides higher accuracy than models containing all data points.

### 3.2. Comparison to Model Predictions

Prediction models were evaluated by comparing measured versus predicted shear strength for the test data presented earlier. Safety factors were set to 1.0 when calculating the predicted shear strength of UHPC beams. Crack angles that were not reported for some tests were assumed based on shear failure photos. The tensile strength that was not reported for some tests was assumed based on direct tension or flexural test results. A conversion factor was used to convert the tensile strength obtained from flexure testing to that obtained from the direct tension test. This conversion factor was found to be 0.377 based on the average of the German guidelines for UHPC [18] and the Swiss standard [19]. The conversion factor is multiplied by the post-cracking flexural tensile strength to get the axial tensile strength and is based on having the neutral axis located at a distance of about 82% of the prism height from the extreme tension surface. Table 1 presents three-point bending tests conducted in the literature to quantify the residual tensile strength of UHPC. It shows that the size of the prism has a significant effect on the measured tensile strength. Average values from this table were used to estimate the residual tensile strength of UHPC in the terms $f_{R,4}$, $f_{ftuk}$, ${Ϭ}_{Rd,f}$, $f_{rr}$, or $f_{t,loc}$ in the five considered prediction models, respectively. For example, for a fiber volume fraction of 2%, $f_{R,4}$ would be 29.6 MPa, whereas for mixes with 2.5% and 1% fiber volume fractions, $f_{R,4}$ would be to use the 40.0 and 2.8 MPa, respectively. For mixes with different fiber volume fractions, values are estimated by interpolation. A prediction example of Tadros et al., 2021 [8], IA1 data point is presented in the Appendix A section to show how the calculation procedure is performed for the five models.

#### 3.2.1. RILEM TC 162-TDF, 2003

**Figure 8:**
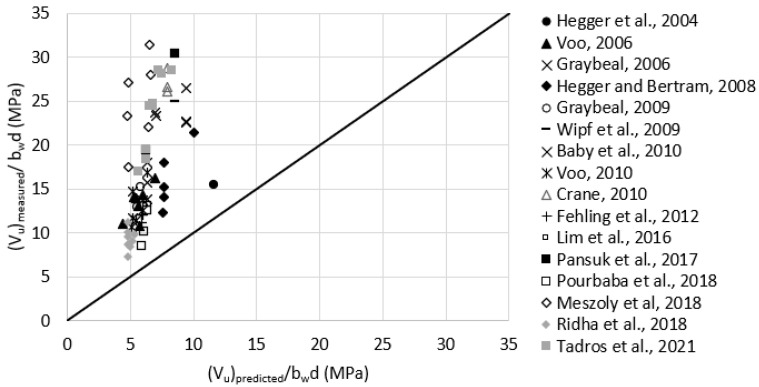
Measured versus predicted shear strength according to RILEM TC 162-TDF, 2003 (Equations (1) and (5)).

Reasonable assumptions for unreported values of $f_{R,4}$ were made according to Table 1. For example, $f_{R,4}$ was assumed as 29.6 MPa for test data having $V_{f}$ = 2.0%. Figure 8 plots the measured versus predicted shear strength using RILEM TC 162-TDF model. The average $\frac{{\left(V_{u}\right)}_{measured}}{{\left(V_{u}\right)}_{predicted}}$ was 2.7, with a standard deviation of 0.88. It can be noticed that the underestimation increases with the increase of the measured shear strength.

#### 3.2.2. *Fib* Model Code, 2010

**Figure 9:**
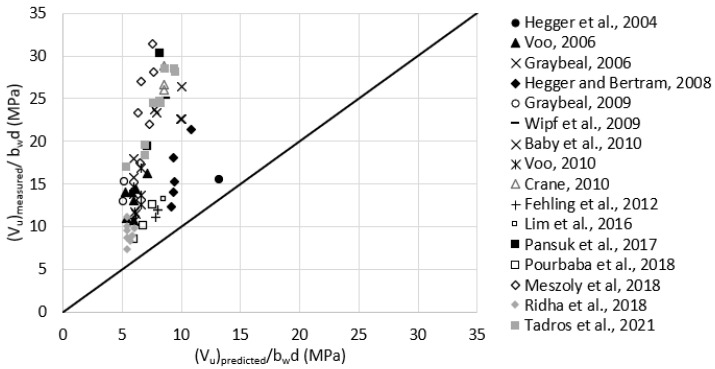
Measured versus predicted shear strength according to the *fib* Model Code, 2010 (Equation (8)).

Reasonable assumptions for unreported values of $f_{ftuk}$ were made according to Table 1. For example, $f_{ftuk}$ was assumed as 11.0 MPa for test data having $V_{f}$ = 2.0%. This assumption is based on Equations (10) and (11) and having the flexural tensile strengths ($f_{R,i}$) according to Table 1. Figure 9 plots the measured versus the predicted shear strength *fib* Model Code. The average $\frac{{\left(V_{u}\right)}_{measured}}{{\left(V_{u}\right)}_{predicted}}$ was 2.4, with a standard deviation of 0.74. It can be noticed that the predicted shear strength is close to what the RILEM TC 162-TDF, 2003 5 provides.

#### 3.2.3. French Standard, NF P 18-710, 2016

**Figure 10:**
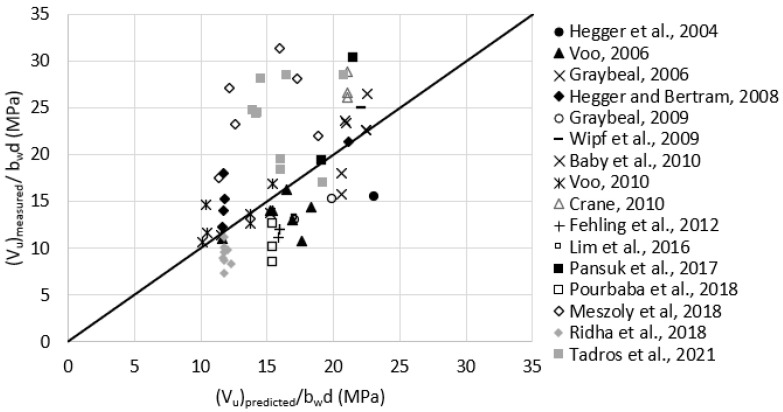
Measured versus predicted shear strength according to French Standard NF P 18-710, 2016 (Equations (13) or (14), and (16)).

Reasonable assumptions for unreported values of ${Ϭ}_{Rd,f}$ were made according to Table 1. For example, ${Ϭ}_{Rd,f}$ was assumed as 11.0 MPa for test data having $V_{f}$ = 2.0%. Figure 10 plots the measured versus predicted shear strength using the French standard model. The average $\frac{{\left(V_{u}\right)}_{measured}}{{\left(V_{u}\right)}_{predicted}}$ was 1.1, with a standard deviation of 0.38. It can be noticed that the data are well distributed around the 45-degree angle line indicating a reasonable consistency in the prediction accuracy.

#### 3.2.4. PCI-UHPC Structures Design Guide, 2021

**Figure 11:**
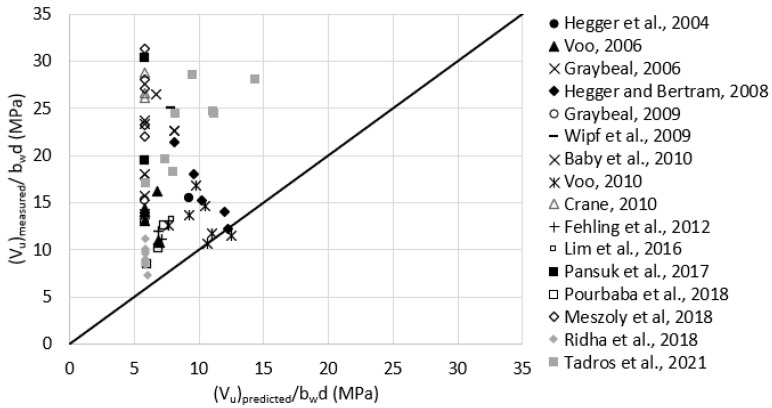
Measured versus predicted shear strength according to PCI-UHPC Structures Design Guide, 2021 (Equation (18)).

The assumed value of $f_{rr}$ was limited to 5.2 MPa as recommended by the model. This value is significantly lower than what is typically achieved by commonly used UHPC mixes, which caused the predicted shear strengths to be significantly underestimated. The crack angle was calculated according to the suggested procedure of the model and was limited to range between 27.6 and 50.0 degrees as recommended. Figure 11 plots the measured versus predicted shear strength using the PCI-UHPC Structures Design Guide model. The average $\frac{{\left(V_{u}\right)}_{measured}}{{\left(V_{u}\right)}_{predicted}}$ was 2.5, with a standard deviation of 1.14. It should be noted that the French Standard takes into account the level of prestressing whereas the PCI-UHPC model does not consider the level of prestressing term for simplicity. The effect of prestressing is only considered in that model when estimating the crack angle.

#### 3.2.5. Draft of AASHTO Guide Specification for Structural Design with UHPC, 2021

**Figure 12:**
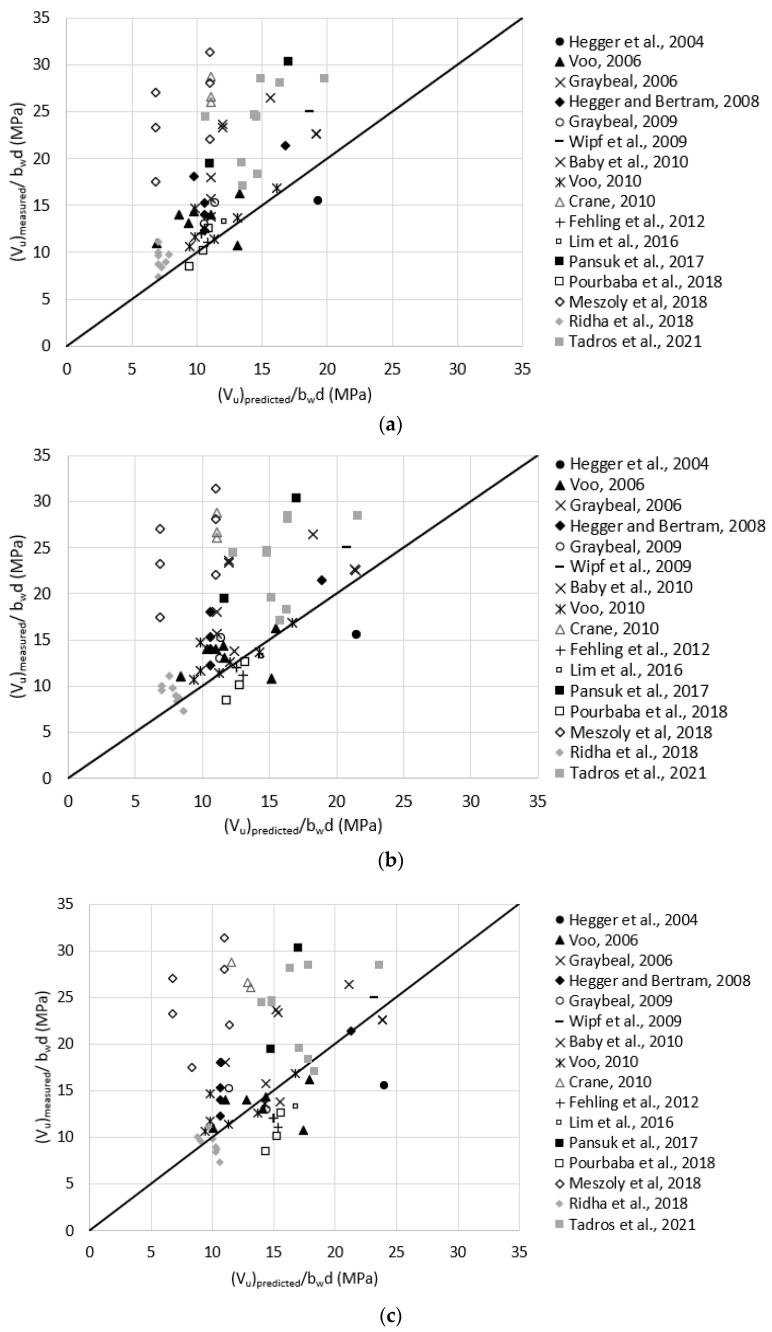
Measured versus predicted shear strength according to Draft of AASHTO Guide Specification for Structural Design with UHPC, 2021; (**a**) ${Ꜫ}_{t,loc}$ = 0.005; (**b**) ${Ꜫ}_{t,loc}$ = 0.007; (**c**) ${Ꜫ}_{t,loc}$ = 0.010 (Equation (22)).

Reasonable assumptions for unreported values of $f_{t,loc}$ were made according to Table 1. For example, $f_{t,loc}$ was assumed as 11.0 MPa for test data having $V_{f}$ = 2.0%. The comparison to this model prediction was done for three cases (a to c) where the localization strain (${Ꜫ}_{t,loc}$) was considered as 0.005, 0.007, and 0.010, respectively, for all test data based on the recommended range of 0.004 and 0.010 [20,21]. Figure 12 plots the measured versus predicted shear strength using Draft AASHTO model for the three cases. The average $\frac{{\left(V_{u}\right)}_{measured}}{{\left(V_{u}\right)}_{predicted}}$ were 1.6, 1.5, and 1.3 with a standard deviation of 0.60, 0.63, and 0.64 for cases a through c, respectively. This indicates that the prediction provided by this model is not very sensitive to ${Ꜫ}_{t,loc}$, and doubling ${Ꜫ}_{t,loc}$ from 0.005 to 0.010 resulted in an increase of 23% of the average prediction. Additionally, the prediction provided by that model is slightly more conservative than the one provided by the French Standard and is closest to the measured shear strength compared to all other prediction models.

## 4. Discussion and Recommendation

Table 2 summarizes the outcomes of model evaluation. The table shows that the French Standard NF P 18-710, 2016 [7] provided the closest estimation of the shear strength followed by the Draft of AASHTO Guide Specification for Structural Design with UHPC, 2021 [9]. The PCI-UHPC Structures Design Guide, 2021 [8], underestimated the predicted shear strength significantly due to assuming a constant conservative value of UHPC tensile strength. The *fib* Model Code, 2010 [6], and RILEM TC 162-TD, 2013 [5], models provided significantly conservative prediction as they were developed for steel fiber-reinforced concrete. The French Standard NF P 18-710, 2016 [7] also provided the least standard deviation, which indicates the highest consistency in the shear strength prediction. Furthermore, the PCI-UHPC and Draft AASHTO models do not consider explicitly the level of prestressing in the fiber contribution term as the French Standard does. The prestressing effect is only considered in the crack angle estimation equations. This is one of the major factors that causes the French Standard to provide higher consistency in the shear strength prediction.

Based on the findings of this study, it is recommended to use the French Standard prediction model to estimate the shear strength of UHPC beams if the closest prediction is required. The PCI-UHPC and Draft AASHTO models can also be used but the designer should be aware of increased conservatism.

## 5. Conclusions

The paper presented five prediction models for the shear strength of prestressed and non-prestressed UHPC beams without transverse reinforcement. Available test data in the literature were used to evaluate these models and identify the key parameters that affect the shear strength of UHPC beams. Despite the limited size of published test data, this investigation yielded the following conclusions:

1. Among the parameters affecting the shear strength of prestressed and non-prestressed beams, the tensile strength of UHPC (${Ϭ}_{Rd,f}$) was found to have a significant positive correlation with the shear strength of UHPC beams.
2. For UHPC beams with a fiber volume fraction of at least 2%, the level of prestressing (${Ϭ}_{cp}$) and longitudinal reinforcement ratio ($A_{l}/b_{w}d$) were found to have a significant effect on the shear strength of prestressed and non-prestressed beams, respectively.
3. The French Standard model provided the closest prediction to the measured shear strength of UHPC beams with the highest consistency of prediction, followed by the Draft of AASHTO model.
4. The PCI-UHPC Structures Design Guide prediction model significantly underestimates the shear strength due to the limitation of UHPC residual tensile strength to 5.2 MPa. This value is significantly smaller than what is typically achieved by the commonly used UHPC mixes. However, the model procedure is much simpler compared to the other models.
5. The RILEM and *fib* Model Code, 2010 prediction models were developed primarily for fiber-reinforced concrete and significantly underestimate the shear strength of UHPC beams.
6. Residual tensile strength of UHPC is an essential parameter in all UHPC prediction models. The method of its determination is not unified between the shear experiments and prediction models. Moreover, the variability in residual tensile strength among specimens is large, which requires several direct tension or flexure tests to yield reliable predictions.

## Figures and Tables

**Table 1:** UHPC residual tensile strength reported in the literature.

Reference	Fiber Volume $\left(V_{f}\right)$	Fiber Length (l_f_) (mm)	Fiber Diameter (Φ_f_) (mm)	Cylinder Compressive Strength $\left({f_{c}}^{\prime })\;(\mathrm{MPa}\right)$	Prism Cross Section (b × h) (mm^2^)	Notch Height (mm)	Span (mm)	Residual Flexural Tensile Strengths $\left(f_{R,i})\;(\mathrm{MPa}\right)$			
								$f_{R,1}$	$f_{R,2}$	$f_{R,3}$	$f_{R,4}$
Prem et al., 2012 [40] (R1 Mix)	2.5%	13	×	180.0	70 × 70	21.0	300	45.5	49.6	42.7	40.0
Prem et al., 2012 [40] (R2 Mix)	2%	13	0.15	170.0	70 × 70	21.0	300	37.2	40.0	37.2	34.5
Yang et al., 2010 [41] (average)	2%	13	0.2	190.9	100 × 100	10.0	300	26.9	30.3	27.6	24.8
Graybeal, 2006 [3] (M2P02)	2%	13	0.2	126.2	50 × 100	25.4	406	22.1	20.7	--	--
Zagon et al., 2016 [42] (average)	1%	10	0.18	141.3	100 × 100	27.0	400	11.7	6.2	4.1	2.8

**Table 2:** Summary of evaluation of prediction models.

Prediction Model	$\mathrm{Average}\;\frac{{\left(V_{u}\right)}_{measured}}{{\left(V_{u}\right)}_{predicted}}$	Standard Deviation
RILEM TC 162-TDF, 2003 [5]	2.7	0.88
*fib* Model Code, 2010 [6]	2.4	0.75
French Standard NF P 18-710, 2016 [7]	1.1	0.38
PCI-UHPC Structures Design Guide, 2021 [8]	2.5	1.15
Draft of AASHTO Guide Specification for Structural Design with UHPC, 2021 [9]—Case (a)	1.6	0.60
Draft of AASHTO Guide Specification for Structural Design with UHPC, 2021 [9]—Case (b)	1.5	0.63
Draft of AASHTO Guide Specification for Structural Design with UHPC, 2021 [9]—Case (c)	1.3	0.64

## Appendix A

**Table A1:** Shear experiments review on prestressed UHPC beams.

Reference	Specimen ID	Specimen Shape	b_w_ (mm)	d (mm)	h (mm)	$V_{f}\;\%$	a/d	A_ps_/b_w_d	$\sigma_{cp}\;(\mathrm{MPa})$	${f^{\prime }}_{c}$ (MPa)	l_f_ (mm)	Φ_f_ (mm)	$\sigma_{Rd,f}$ (MPa)	θ	V_u_ (kN)	$\left(\frac{V_{u}}{b_{w}d}\right)$ (MPa)
Hegger et al., 2004 [24]	1	I-beam	70	250	300	2.5	5.4	6.80%	26.9	192	13	0.15	12.1	31	271.3	15.9
Voo et al., 2006 [23]	SB2	I-beams	50	600	650	2.5	3.3	2.70%	14.5	160	13 (Type I) 30 (Type II)	0.20 (Type I) 0.50 (Type II)	9.8	34	496.8	16.6
	SB3								6.9	149			8.6	32	427.9	13.8
	SB4					1.25				164			5.5	26	336.3	11.0
	SB5					2.5				171			9.7	29	439.9	14.5
	SB6									157			9.3	24	330	11.0
	SB7									169			8.8	21	399.9	13.1
Graybeal, 2006 [27]	28S	AASHTO Type II	155	803	910	2.0	2.5	1.90%	9.0	193	13	0.20	11.0	40	1707.6	13.8
	24S						2.8						11.0	28	2232.5	17.9
	14S						2.3						11.0	25	1946.9	15.9
Hegger and Bertram, 2008 [25]	T1a	I-beams	60	318	400	0.9 2.5 0.9	3.8	4.60%	24.8	144	18	0.15	5.0	30	234	12.4
	T1b									165			5.0	30	266.9	13.8
	T3b									162	9		11.0	30	407.9	21.4
	T4a									167	18		5.0	30	343.8	17.9
	T4b						4.4			174			5.0	30	290.9	15.2
Graybeal, 2009 [28]	P 2-21S	Pi-girders	170	750	838	2.0	2.9	1.73%	6.2	229	13	0.20	11.4	32	1912.6	15.2
	P4-57SH						2.4						10.6	35	1628.0	13.1
Wipf et al., 2009 [29]	1	I-beams	115	927	1067	2.0	2.5	5.6%	21.4	153	13	0.20	11.7	25	2642.1	24.8
Baby et al., 2010 [31]	Beam 1-A	I-beams	65	305	380	2.5	2.5	4.10%	17.2	185	20	0.30	12.0	30	441.2	22.1
	Beam 1-A-bis									192			12.0	30	440.4	22.1
	Beam 1-B					2.0				201	13	0.20	12.0	30	515.5	26.2
Voo et al., 2010 [30]	X-B1	I-beams with Symmetric Prestressing	50	620	650	1.0	3.2	2.60%	15.2	125	15	0.20	4.4	26	330.0	10.4
	X-B2									126			5.3	24	355.0	11.0
	X-B3									135			4.6	22	362.1	11.7
	X-B4						2.5			122			4.6	26	455.5	14.5
	X-B5						3.5			140			6.6	24	422.6	13.1
	X-B6						4.5			140			6.6	24	390.5	12.4
	X-B7					1.5	2.5			122			7.8	29	521.3	16.6
Crane, 2010 [15]	1-2	Bulb-Tee Girders	101	720	835	2.0	3.4	3.90%	16.5	200	13	0.20	11.0	26	1917.1	26.9
	2-1													23	2072.8	29.0
	3-1													25	1877.1	26.2
Tadros et al., 2021 [8]	IA1	I-beams	76.2	734.0	863.6	2%	2.9	6.5%	18.0	154.6	13	0.20	11.0	26.8	1596.9	28.3
	IA2									120.1			6.9	30.8	1383.4	24.8
	IA3									126.3			6.9	28.3	1370.0	24.8
	IA8									125.6			8.3	28.0	1596.9	28.3
	IA13		50.8					9.8%	19.3	126.3			7.6	32.0	1049.8	28.3
	IA14		101.6					4.9%	16.9	126.3			6.9	28.0	1823.8	24.2
	DIB—Test 1	Decked I-Beam	100	924.0	1000		2.7	2.2%	6.4	138.0			10.4	28.0	1579.1	17.3
	BX-1	Box Section	152.4	406.4	457.2		2.8	3.5%	9.8	136.6			8.3	25.0	1214.4	19.3
	BX-2														1138.7	18.4
		Maximum	170	927	1067	2.5	5.4	9.80%	26.9	229	30	0.50	12.1	40	2642.1	29.0
		Minimum	50	250	300	0.9	2.3	1.73%	6.2	122	9	0.15	4.4	22	234	10.4

**Table A2:** Shear experiments review on non-prestressed UHPC beams.

Reference	Specimen ID	Specimen Shape	b_w_ (mm)	d (mm)	h (mm)	$V_{f}$ %	a/d	$\frac{A_{l}}{b_{w}d}$	${f^{\prime }}_{c}$ (MPa)	l_f_ (mm)	Φ_f_ (mm)	${Ϭ}_{Rd,f}$ (MPa)	θ	V_u_ (kN)	$\left(\frac{V_{u}}{b_{w}d}\right)$ (MPa)
Voo et al., 2006 [23]	SB1	I-beams	50	600	650	2.5	3.3	2.70%	161	13(Type I) 30 (Type II)	0.20 (Type I) 0.50 (Type II)	11.0	37	430.1	13.8
Baby et al., 2010 [31]	Beam 3-A	I-beams	65	305	380	2.5	2.5	4.80%	185	20	0.30	12.0	30	461.3	23.5
	Beam 3-B					2.0	2.5	4.80%	201	13	0.20	12.0	30	455.0	22.8
Fehling et al., 2012 [33]	Q-F1-2	Shear span was I-shaped	30	295	320	1.0	4.1	6.60%	201	13	0.18	8.7	30	108.1	12.4
	Q-F1-3								207			8.7	30	108.1	12.4
	Q2-F1-1								185			8.7	30	100.1	11.0
Lim et al., 2016 [26]	SB1	Rectangular Beams	150	240	290	1.5	2.8	7.30%	167	16 and 19	0.20	9.6	27	475.9	13.1
Pourbaba et al., 2018 [34]	B35	Rectangular Beams	152	56	76	1.5	2.7	5.60%	125	13	0.18	9.4	32	105.9	12.4
	B36							4.00%				9.4	32	85.0	9.7
	B37							2.70%				9.4	32	71.2	8.3
Pansuk et al., 2017 [36]	NS08	I-beams	50	350	400	0.8	2.9	5.50%	141	13	0.20	11.0	30	339.8	19.3
	NS16					1.6			140			17.0	38	531.1	30.4
Meszoly et al., 2018 [37]	B19	I-beams	60	295	350	2.0	3.7	5.06%	152	15	0.20	11.0	30	396.8	22.8
	B20					1.0			154			6.8	29	419.0	23.5
	B24								166			6.8	34	314.9	17.9
	B25					2.0			179			11.0	33	504.8	28.3
	B29								177			6.8	31	487.1	27.6
	B30					2.0			169			11.0	36	564.9	31.7
Ridha et al., 2018 [38]	B5	Rectangular Beams	100	112	140	2.0	3.5	2.90%	110	13	0.20	7.0	32	82.3	7.6
	B6											7.0	32	107.6	9.7
	B7											7.0	32	112.5	10.4
	B10						2.5					7.0	32	125	11.0
	B11						3.0					7.0	32	97.4	9.0
	B16						3.5		125			7.3	32	93.9	8.3
	B17								142			7.6	35	101.0	9.0
	B18								151			7.8	35	109.9	9.7
		Maximum	152	600	650	2.5	4.1	7.30%	207	30	0.50	17.0	38	564.9	30.4
		Minimum	30	56	76	0.8	2.5	2.70%	110	9	0.15	6.8	27	71.2	7.6
